# Deciphering the Properties of Nanoconfined Aqueous Solutions by Vibrational Sum Frequency Generation Spectroscopy

**DOI:** 10.1021/acs.jpclett.2c03409

**Published:** 2023-01-30

**Authors:** Banshi Das, Sergi Ruiz-Barragan, Dominik Marx

**Affiliations:** † Lehrstuhl für Theoretische Chemie, Ruhr-Universität Bochum, 44780Bochum, Germany; ‡ Departament de Fisica, Universitat Politecnica de Catalunya, Rambla Sant Nebridi 22, 08222 Terrassa, Barcelona, Spain

## Abstract

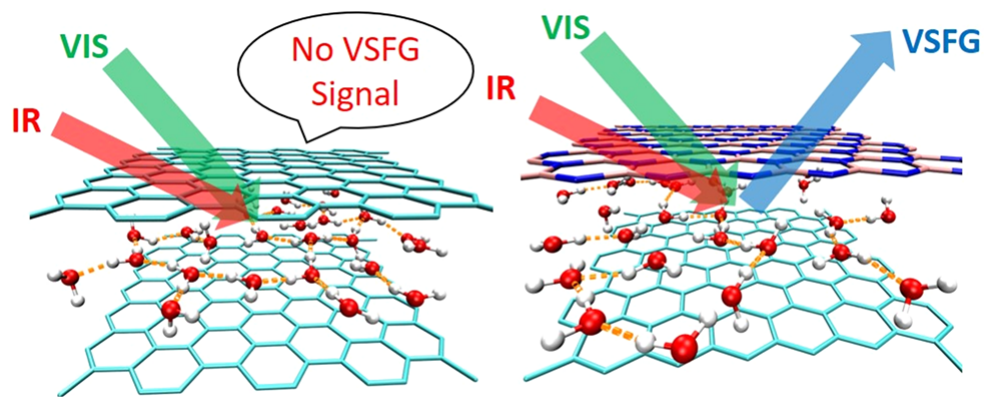

When confined between walls at nanometer distances, water exhibits surprisingly different properties with reference to bare interfacial water. Based on computer simulations, we demonstrate how vibrational sum frequency generation (VSFG) spectroscopy can be used–even with very mild symmetry breaking–to discriminate multilayer water in wide slit pores from both bilayer and monolayer water confined within molecularly narrow pores. Applying the technique, the VSFG lineshapes of monolayer, bilayer, and multilayer water are found to differ in characteristic ways, which is explained by their distinct density stratifications giving rise to different H-bonding patterns in the respective solvation layers.

Nanoconfined water and aqueous solutions increasingly attract much attention across the disciplines in view of both their peculiar properties and prospects toward technological applications.^1−7^ In particular, transport properties, dielectric responses, and chemical equilibria have been shown repeatedly to be enormously affected depending on the topology of the confinement, the extent of the confinement, and the nature of the confining materials.^8−18^ Carbon nanotubes have been studied to nanoconfine water in cylindrical pores, whereas nanoconfinement of aqueous solutions in slit pores with controlled thicknesses down to the subnanometer scale became possible recently.^10,13,14,16^ Such nanochannel devices can be fabricated using graphite or graphene walls (GRA),^10,13,16^ other layered materials such as hexagonal boron nitride (HBN) or MoS_2_,^13,16,19^ as well as hybrid combinations of those.^14^ In the extreme confinement regime, corresponding to interlayer distances of 1 nm and less, water and aqueous solutions are squeezed into bilayers and even monolayers that form highly stratified two-dimensional H-bond networks as reviewed recently.^7^

In stark contrast to bare (or open) interfaces, where interfacial water is in contact with bulk water for sufficiently thick water films on a surface, strongly confined water will be influenced by the two confining surfaces (walls) in the limit of narrow slit pores, eventually leading to monolayer or bilayer water lamellae. While dielectric measurements only capture the collective response of the entire slit pore,^9,12,14,18,20^ thus not providing molecular-level insights, vibrational sum frequency generation (VSFG) spectroscopy^21,22^ in the mid-IR region is an utmost surface-sensitive probe of the local H-bonding environment of water close to interfaces. Indeed, in the context of standard interfacial water, VSFG has been demonstrated to be an excellent method to decipher the details of the interfacial H-bonding characteristics.^23−34^ Despite this great interface-sensitivity, its use is hitherto unknown in the realm of nanoconfined liquids.

Here, we apply this second-order nonlinear optical technique to nanoconfined water and compute the VSFG spectral responses of water lamellae of varying thickness within suitably symmetry-broken slit pores–finding that rather small differences in wall material are sufficient. Next, we demonstrate that VSFG spectroscopy is able to reveal the very characteristic molecular changes that are imprinted by confining water. Upon molecular decomposition of these spectra, we provide unprecedented insights into the peculiar H-bonding pattern of highly stratified monolayer and bilayer water in mixed GRA–HBN slit pores as seen by VSFG—much like those hitherto extracted from VSFG applied to unravel interfacial water. Finally, we introduce a novel analysis technique that can be readily applied to experimental VSFG spectra of nanoconfined aqueous solutions to connect the measured confinement-induced spectral signatures to the H-bonding pattern also in more complex slit pore setups.

*Slit Pore Setup and Simulations*. We have used coplanar GRA and HBN sheets to create slit pores of different interlayer distances *d*_int_. They have been filled with an increasing number of water molecules (see Table S1) in order to generate confined water lamellae from the monolayer and bilayer regime (XS and S) to multilayers (M and L) to the XL pore featuring a bulk-like density in its innermost region, see Figure 1. The equilibrium values *d*_int_ have been determined at 300 K by carrying out fixed normal pressure simulations corresponding to 1 bar using our piston approach,^18^ see Table S1. All observables, notably the VSFG spectra, have been computed from statistically independent microcanonical (NVE) trajectories initiated from canonical (NVT) simulations to ensure rigorous calculation of time-correlations functions at constant temperature and pressure conditions according to eq 1. All simulations have been carried out using force field molecular dynamics, see section S1.

**Figure 1:**
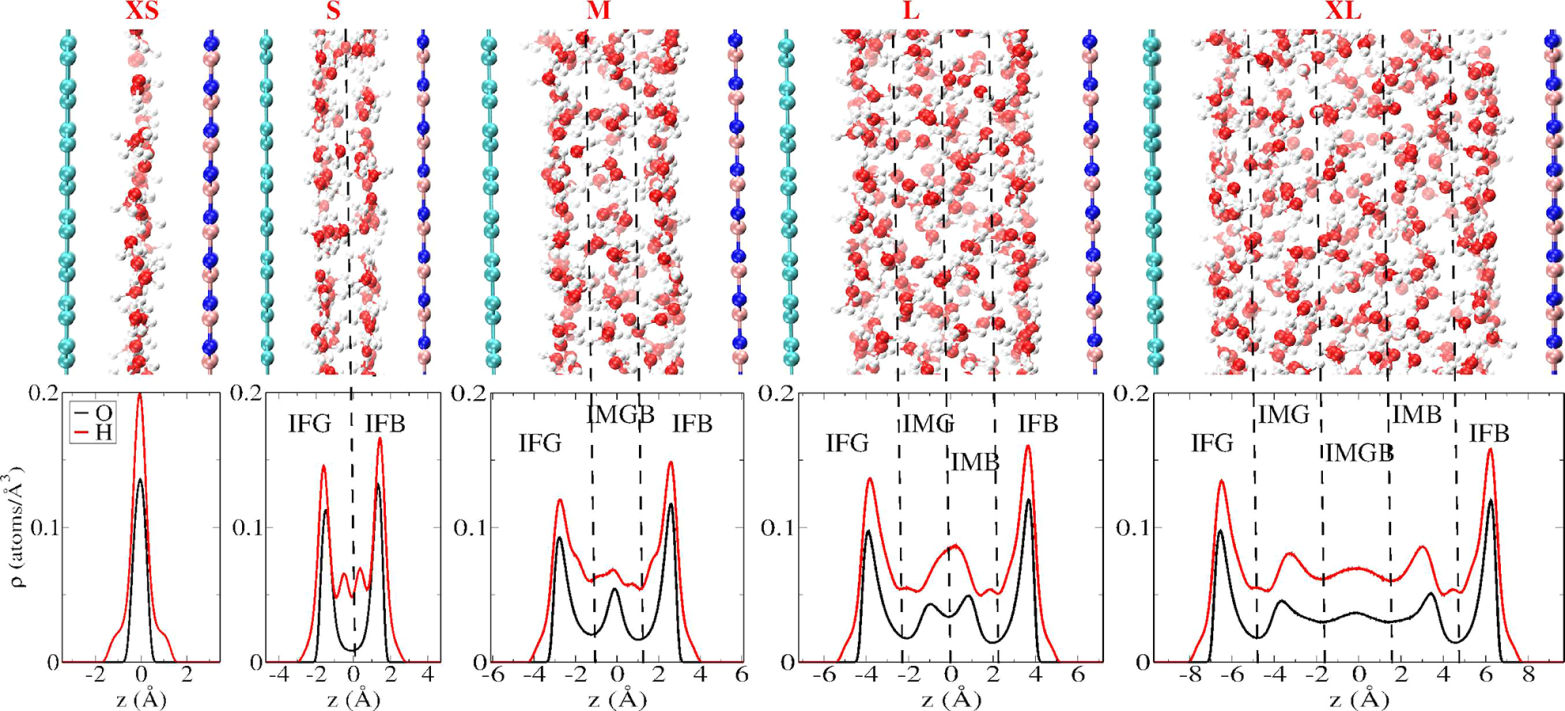
Number density profiles of water in GRA–HBN slit pores along the surface normal (*z*-axis) in terms of oxygen (black) and hydrogen (red) densities for the XS to XL setups (see text) with corresponding representative configuration snapshots in the upper panels; C, B, and N atoms of GRA (left walls) and HBN (right walls) are shown in cyan, orange, and blue, respectively; the *z*-axis points from GRA (left) to HBN (right) walls. The different interfacial (IF) and intermediate (IM) solvation layers are determined by the minima of ρ_O_(*z*) as marked using vertical black dashed lines.

*Computational Spectroscopy*. In VSFG experiments,^21,22^ input IR light with variable frequency ω and input visible light at fixed frequency ω_VIS_ coherently interact with the material and thereby generate an interface-sensitive VSFG signal at frequency ω_VSFG_ = ω + ω_VIS_ due to the symmetry-breaking influence of interfaces. In the context of aqueous interfaces, the resonant part of the VSFG line shape function can be computed approximately for the vibrationally isolated O–H chromophore (of HOD molecules in liquid D_2_O) within a mixed quantum/classical approach,^35−37^
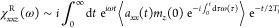
1where *xxz* refers to the SSP polarization combination of the output and input signal, *a*_*xx*_ is the *xx*-component of the transition polarizability, *m*_*z*_ is the *z*-component of the transition dipole, ω is here the transition frequency for the 0–1 transition of the O–H vibrational chromophore, and the population relaxation lifetime of the excited state vibration *T*_1_ is taken from experiment. We have adopted^38^ the well-established electronic structure/molecular dynamics (ES/MD) method^35,39,40^ to evaluate eq 1; see section S2 for background and computational details. This efficient combination of parametrized techniques has been shown to provide semiquantitative insight into VSFG spectra which allows for qualitative interpretation.^35,41^

In an effort to validate the particular approach used for the present investigation, we explicitly compare in section S2 the VSFG spectra calculated from the parametrized ES/MD approach to those obtained from sophisticated *ab initio* MD simulations^42^ for HBN–water, GRA–water, and water–air interfaces. The relative VSFG spectral shift as depicted in Figure S1 (which will be shown below to play a crucial role in case of water confined in slit pores) is found to be very similar compared to the AIMD benchmark result.^42^ In addition, comparison of the experimental VSFG spectrum^43^ of the water–air interface in Figure S2 with the spectra calculated using the two vastly different computational methods clearly shows the ability of the ES/MD approach adopted herein to generate VSFG spectra of these bare interfaces at a similar accuracy level as that provided by the state-of-the-art AIMD technique.

*Water Stratification across Asymmetric Slit Pores*. The density profiles of water normal to the confining GRA and HBN walls depicted in Figure 1 appear at first glance to be rather similar to what is well-established now for water that is hosted within GRA–GRA slit pores at very similar interlayer distances.^15,18^ These setups cover the crossover from monolayer and bilayer water in the XS and S slit pores, respectively, to multilayer lamellae (M and L) and finally to less strictly confined water that shows bulk-like densities in the central region (see IMGB in the XL setup). In stark contrast to the symmetric GRA–GRA slit pores, the density profiles of the GRA–HBN systems are not symmetric across the pores, i.e., along the *z*-axis, see Figure 1. The modest skewness is imprinted by the slightly different H_2_O···GRA versus H_2_O···HBN noncovalent interactions—the latter being little stronger leading to more pronounced maxima that are pulled closer to HBN. Thus, unlike in symmetric confinement, there are two different kinds of interfacial solvation layers (IF), namely IFG and IFB denoting first shell solvation water close to the GRA and HBN walls, respectively. Similarly, the intermediate solvation layers (IM) are structured differently if they are closer to either GRA or HBN, i.e., IMG and IMB; note that the density profile of the bulk-like region IMGB in the largest pore XL appears already quite symmetric. As will be demonstrated, the resulting slight asymmetry suffices to break the symmetry: The resulting VSFG spectra are found to be very sensitive probes that provide molecular insights into nanoconfinement effects on H-bonding in narrow asymmetric slit pores where the shape of the water lamellae results from an interplay of the different confining materials.

*VSFG Fingerprints of Confined Water.* The VSFG spectral responses of water within GRA–HBN slit pores of increasing thickness, from monolayer (XS) to bilayer (S) to multilayer (M and L) and beyond (XL), are collected in Figure 2. Indeed, one finds a significant and well-structured VSFG response of these water lamellae (colored lines) even given the rather mild asymmetry established upon confining water using only sightly different wall materials as quantified at the level of the water density profiles in Figure S4. As internal reference, the VSFG response from bilayer water confined within the symmetric GRA–GRA slit pore S is confirmed to be vanishingly small (circles); the same is found for monolayer water (XS) in Figure S3. Thus, even the weak centrosymmetry breaking induced by the difference of HBN versus GRA walls suffices to make confined water VSFG-active.

**Figure 2:**
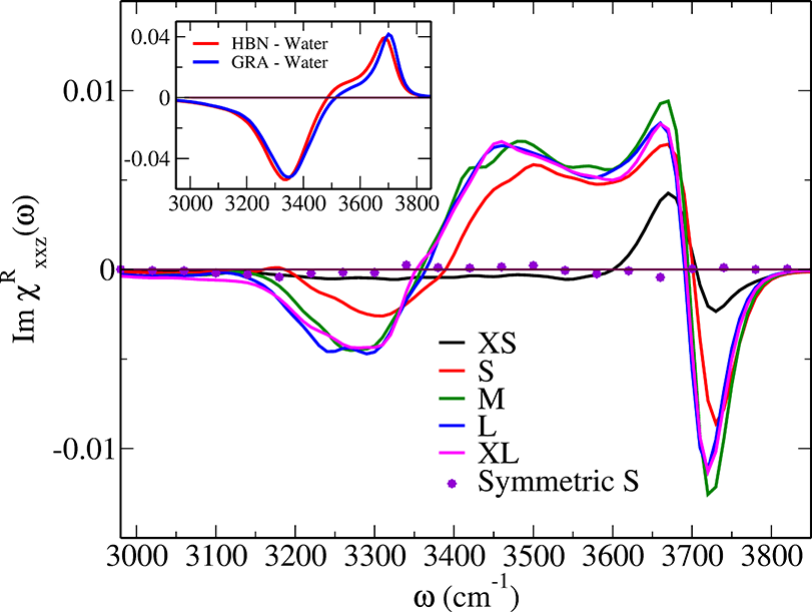
Imaginary part of the VSFG spectral responses according to eq 1 computed for water lamellae confined within asymmetric GRA–HBN slit pores of different interlayer distances from XS to XL (colored lines, see text) as well as for the symmetric GRA–GRA system S (circles) and for water in contact with GRA or HBN surfaces are shown in inset (see section S5 for the respective computational approach). The zero intensity line is shown by a thin solid line.

Already at the level of visual inspection, one can identify three different classes of spectra that might indicate different confinement regimes. The water monolayer within the ultranarrow XS pore exclusively features signals at high frequencies of about 3700 cm^–1^ that are known from dangling (free) O–H bonds at water/vapor or water/hydrophobic interfaces,^44−46^ whereas no VSFG response is found below 3600 cm^–1^. Grossly speaking, the three wider slit pores provide qualitatively similar VSFG lineshapes that extend from the free O–H regime down to 3100 cm^–1^, thus covering the broad O–H stretching band known from H-bonding in liquid water. Interestingly, the bilayer S yields a VSFG spectrum that has more similarities in the shape to those of the more weakly confined systems (M, L, and XL) than to that of the monolayer XS, yet characteristic peak shifts and intensity differences are observed at typical H-bonding frequencies.

*Molecular Deconvolution of VSFG Spectra*. The VSFG responses reported in Figure 2 have grossly different lineshapes in comparison to those known for typical water–air or hydrophobic water interfaces. This is evidenced when comparing those in the inset computed for hydrophobic water interfaces at both GRA and HBN surfaces using the same techniques (see section S5). It is well-known that their shape is dominated by two main peaks,^26,41^ namely the low frequency peak around ≈3400 cm^–1^ corresponding to the so-called ”down” oriented (toward bulk water) O–H bonds that donate an H-bond themselves, thus being subject to H-bonding within the aqueous phase, and the high frequency peak at ≈3700 cm^–1^ that corresponds to the ”up” oriented (toward the air/hydrophobic surface) O–H bonds, also denoted as dangling or free O–H bonds which are not involved in H-bonding as sketched in Figure 3(a). While the former ones generate a negative signal within that standard convention, the latter are characterized by positive intensity thus generating the typical VSFG line shape presented in the inset of Figure 2 for separate GRA–water and HBN–water interfaces.

**Figure 3:**
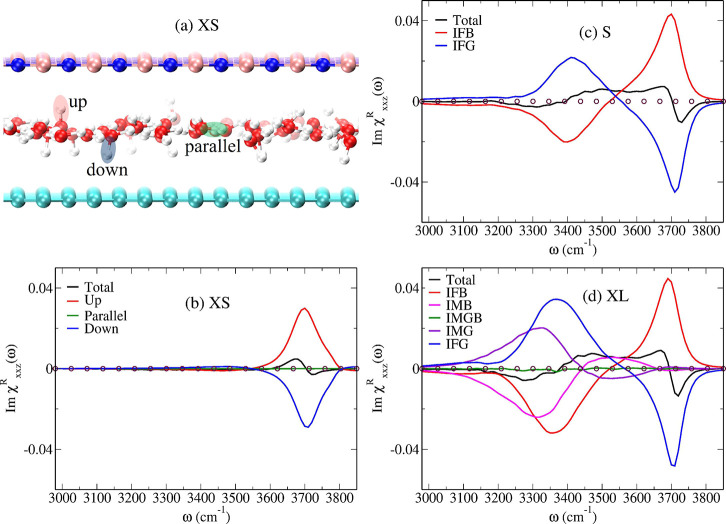
(a) Representative configuration snapshot of the monolayer slit pore XS where up, down, and parallel oriented O–H bonds serving as VSFG chromophores are highlighted; the *z*-axis points from graphene (bottom) to the boron nitride (top) wall. Panels (b) to (d) report the spectral deconvolutions of the total VSFG signal for the slit pores as indicated in the insets and explained in the text; note that the green lines in (b) and (d) stay close to zero. The zero intensity line is shown by open circles in panels (b) to (d).

To understand the origin of the vastly different VSFG lineshapes of strongly confined water compared to those known from interfacial water, it is instructive to decompose the total spectral response into partial responses from different subensembles of the whole system. This is commonly realized^47,48^ by introducing a step function θ_α_(*t*), being unity if the O–H chromophore is in region α at time *t* and zero otherwise, into the time-correlation function θ_α_(0)*a*_*ij*_(*t*)*m*_*k*_(0) within eq 1. The resulting partial VSFG responses Im  are compiled in Figure 3 and Figure S5 for all asymmetric slit pores.

For XL (and also L and M), the partial VSFG signals from confined water in the interfacial layers IF close to either GRA or HBN (i.e., IFG and IFB, respectively) are very similar and, more importantly, clearly yield the typical features of the total VSFG spectra of GRA–water and HBN–water interfaces as shown in the inset of Figure 2; note the good agreement of our GRA–water spectrum with what is known from experimental and computational literature.^42,49,50^ Qualitatively speaking, one peak is observed at low frequencies corresponding to those O–H oscillators that donate H-bonds to water molecules in the adjacent intermediate layer (i.e., IMG and IMB) and one in the high frequency region due to dangling O–H bonds pointing toward either GRA or HBN, see Figure 3(d) and Figure S5 for L and M. Due to the convention we adopted here as depicted in Figure 3(a), the free O–H peak from IFB water resembles that found at water–air or hydrophobic water interfaces and, conversely, IFG water has similar features with an inverted sign. In the widest slit pores XL and L, water in the intermediate layers (IMG and IMB) only features the pronounced H-bonded O–H band extending from roughly 3100 to about 3400 cm^–1^, whereas the free O–H stretching peak close to 3700 cm^–1^ is completely missing. Finally, water in the central region of the respective slit pores, i.e., IMGB in setups M and XL, only contributes a rather weak background (subject to slight intensity modulations) to the total VSFG signal, see Figure 3(d) and Figure S5. Overall, we conclude that the total VSFG spectra of such moderately to weakly confined water subject to multilayer stratification (i.e., M, L, XL) are shaped by O–H resonances stemming from molecules in up to the second solvation layer of the two different walls, graphene and boron nitride. This also explains why the total VSFG spectra of multilayer water is virtually identical as found in Figure 2.

In stark contrast, the most confining slit pore XS hosting a water monolayer does not bear any resemblance to the multilayer systems at the level of its VSFG spectrum, see Figure 3(b). Here, the molecular reason is found by decomposing the total response in terms of the orientation of the O–H bonds with respect to the surface normal as illustrated in Figure 3(a). The up, down, and parallel orientation of the O–H oscillators in XS provide positive, negative, and zero signal as qualitatively expected, see Figure 3(b). Both, the positive and negative responses do originate from the dangling O–H oscillators which point toward the two walls. However, the up-oriented signal is influenced by HBN which interacts a bit more strongly with water compared to GRA, hence that peak is slightly red-shifted with reference to the signal from the down-oriented free O–H bonds close to GRA; note that in the case of the symmetric GRA–GRA slit pore XS the VSFG signals due to up- and down-dangling O–H bonds exactly cancel within noise as shown in Figure S3. It is thus the enormous but not complete cancellation of the only marginally frequency-shifted up- and down-oriented partial contributions stemming from the free O–H bonds that generates at asymmetric confinement conditions the very distinct shape of the total VSFG response of monolayer water in the asymmetric XS slit pore–including its unusual high-frequency feature.

The bilayer slit pore S is very different from the monolayer limit; compare Figure 3 panels c and b. In addition to the positive and negative high-frequency peaks due to the up- and down-oriented free O–H bonds in the two first solvation layers, IFB and IFG, there are broad antiphase signals centered around 3400 cm^–1^ due to H-bonded O–H oscillators. Due to the absence of intermediate layer water, the slightly different extent of cancellation of the responses from IFB and IFG layers (as compared to that of multilayer systems) provides the very distinct line shape of the total VSFG signal of bilayer water.

*Coupled versus Uncoupled Interfaces*. The total VSFG response from water lamellae within slit pores is a combined effect of the responses stemming from the two confining surfaces, here from the GRA and HBN walls. Is there a way to spectroscopically determine the (de)coupling of the two interfaces? The answer is given based on Figure S6(c): The VSFG spectra of water in the M, L and XL slit pores are very similar to the VSFG difference spectrum from the two uncoupled surfaces. Thus, multilayer water in such moderately to weakly confining slit pores is essentially a superposition of interfacial water close to the two confining walls. However, bilayer and monolayer water in the molecularly narrow slit pores S and XS, respectively, is distinctly different from multilayer water in all wider pores, recall Figure 2. Hence, the resulting VSFG spectral differences are a measure of the extent of the coupling of the two walls across the confined water lamellae.

Based on the detailed molecular understanding of the cancellation effect of the responses from the two opposite surfaces in the overall VSFG signal of confined water lamellae, we now introduce a spectral decomposition technique that allows us to readily assign nanoconfinement effects on VSFG spectra. It exclusively relies on approximately representing the VSFG spectra of the two bare interfaces (HBN–water and GRA–water) and of the slit pores in terms of Lorentzians as detailed in section S6. Since this does not require any molecular dissection analyses such as those presented above, the technique can be readily applied also to purely experimental data. For moderately to weakly confined water, i.e., for slit pores M to XL, all Lorentzian oscillators are found to have very similar contributions to the overall VSFG spectra as those that correspond to the superposition of the two decoupled (bare) interfacial water layers. In stark contrast, the spectral density corresponding to the H-bonded region is dramatically reduced for bilayer water S, and it becomes essentially fully suppressed in the monolayer limit. Instead, the VSFG response of the monolayer lamella XS is seen to be entirely dominated by dangling (free) O–H bonds. Given that these findings agree with the sophisticated molecular analyses, the same decomposition technique could be readily applied to measured VSFG spectra to experimentally detect and analyze confinement effects.

*Conclusions and Outlook*. We have demonstrated how VSFG spectroscopy, well known to be a very successful surface-sensitive technique to elucidate interfacial water, can be deployed to investigate nanoconfined water in narrow slit pores. It is found that the symmetry breaking generated when using only slightly different wall materials, here GRA and HBN, suffices to generate a VSFG response able to provide the molecular signatures of H-bonding in ultrathin water films—even if subject to slight asymmetries only. In particular, we show at the molecular level how VSFG discriminates multilayer water in sufficiently wide slit pores from both monolayer and bilayer water confined within very narrow pores based on dangling O–H and H-bonded spectral contributions. In addition, we introduce a decomposition technique for experimental VSFG spectra that allows one to not only detect the presence of monolayer water in slit pores as well as the buildup of multilayer lamellae, but also to quantify the distinct dangling O–H and H-bonded contributions of nanoconfined water. Although experimental realizations of such VSFG experiments are certainly challenging, their prospects to investigate Janus-confined water,^51^ e.g., realized by using hydrophobic and hydrophilic confining walls among many other choices, seems compelling. All this will extend considerably the impact of VSFG spectroscopy from interfacial to nanoconfined liquids, in particular when using advanced or even functionalized wall materials in slit pore setups.

## Supporting Information Available

Details of our simulation protocol, VSFG spectral calculation and validation, quantification of the asymmetry in GRA–HBN slit pores, definition of different layers in the asymmetric confinement, calculation of difference VSFG spectrum, spectral decomposition technique using Lorentzian fittings (PDF)
