# Supplementary material for: Deciphering the Properties of Nanoconfined Aqueous Solutions by Vibrational Sum Frequency Generation Spectroscopy
Source: J Phys Chem Lett. 2023 Jan 30;14(5):1208–13. doi: 10.1021/acs.jpclett.2c03409 (PMC9923734; doi:10.1021/acs.jpclett.2c03409)
Supplement: Supplementary file 1 — jz2c03409_si_001.pdf [file jz2c03409_si_001.pdf]

Supplementary Information to

Deciphering the Properties of Nanoconfined Aqueous Solutions by Vibrational Sum Frequency Generation Spectroscopy

Banshi Das,[*,†] Sergi Ruiz-Barragan,[†,‡] and Dominik Marx[†]

Lehrstuhl für Theoretische Chemie, Ruhr-Universität Bochum, 44780 Bochum, Germany

E-mail: banshi.das@rub.de

[*]To whom correspondence should be addressed

[†]Lehrstuhl für Theoretische Chemie, Ruhr-Universität Bochum, 44780 Bochum, Germany

[‡]Departament de Fisica, Universitat Politecnica de Catalunya, Rambla Sant Nebridi 22, 08222 Terrassa, Barcelona, Spain

1. Simulation Details

Based on our earlier work on the dielectric properties of symmetrically confined water confined between two parallel graphene (GRA) sheets,[1] we designed for the present purpose asymmetric GRA–HBN slit pores by replacing one GRA wall by a hexagonal boron nitride (HBN) sheet. Compared to the previous study, we considerably increased the lateral extent of the slit pores to $(x, y) = (34.7484, 34.3920)$ Å, now using 448 carbon atoms for the GRA sheets (instead of only 122) and, correspondingly, 224 boron plus 224 nitrogen atoms for the HBN walls. The fixed interlayer distance (d_{int}) between the GRA and HBN planes together with the number of water molecules (N_{Water}) has been determined as follows in order to generate a set of five representative confinement regimes from monolayer (XS: extra-small) to bilayer (S: small) to multilayer (M: medium, L: large, and XL: extra-large) water lamellae, see Table S1. These GRA–HBN slit pores compare closely in their interlayer distances and water density profiles to the corresponding set of symmetric GRA–GRA slit pores from Ref. 1, compare Table S1 in the present ESI and Fig. 1 in the present main text to Table S1 in the ESI and Fig. 3 of Ref. 1, respectively.

Table S1: Interlayer distances (in Å) of the five slit pore setups and corresponding number of water molecules in the respective lamellae.

System	XS	S	M	L	XL
d_{int} (Å)	6.68	9.45	11.96	14.20	19.53
N_{Water}	108	216	320	420	624

To initiate the procedure leading to the final setups reported in Table S1, a supercell with $(x, y) = (34.7484, 34.3920)$ Å and a z-dimension of 60 Å has been setup where H_2O molecules have been inserted one by one, starting from 40 up to 640 water molecules. The "rigid piston" approach introduced in Ref. 1 (as explained in the ESI Section I.E therein) has been applied with an excess normal (perpendicular) pressure of 0.3 kbar (to establish the correct bulk water density, see ESI Section I.E.2) together with Nosé–Hoover

thermostatting at 300 K (using a time constant of 0.04 ps) for 4 ns for each system setup. Within these constant normal pressure simulations, the initially very large interlayer separation quickly decreases before it starts to fluctuate around a well–defined average value for the given fixed number of intercalated water molecules present within the respective slit pore. The corresponding final interlayer distances d_{int} which are reported in Table S1 (and used in the subsequent "frozen piston" simulations[1] where the interlayer distances and thus the slit pore volume are kept constant by freezing all atoms in the perfectly coplanar GRA and HBN sheets in space as explained in the ESI Section I.E.4 therein) have been obtained by taking the average after excluding a 1 ns initial equilibration period for each rigid piston simulation. After fixing d_{int}, each slit pore system is furthermore equilibrated in the canonical ensemble for another 2 ns. Each of these NVT simulation of the XS, S, $\cdots$ XL slit pore setups with the number of water molecules reported in Table S1 is then continued to generate a set of 20 initial condition by saving the trajectory every 80 ps. Subsequently, each of those is continued in the microcanonical ensemble for 500 ps, i.e. after switching off thermostatting, to generate 20 statistically independent NVE trajectories that allow us to rigorously compute time-correlation functions and thus proper VSFG spectra at 300 K (obtained as the average of all 20 independent spectra obtained from the NVE runs) as explained in the next sections.

Finally, in an effort to compare the VSFG response of the asymmetric GRA–HBN slit pores to the theoretically expected nil response of the symmetric GRA–GRA counterparts (which is nevertheless numerically nonvanishing due to finite sampling statistics leading to spectral noise), another 20 independent NVE trajectories have been generated for the symmetric GRA–GRA bilayer system S following the aforementioned protocol in order to compare one–to–one to the finite VSFG spectrum of the corresponding GRA–HBN bilayer pore.

For water, the SPC/E model[2] has been used whereas the force fields to describe the water/wall interactions for the HBN and GRA sheets are taken from previous work.[3,4] All

these force field simulations have been performed using the `CP2k` simulation package.[5,6]

2. VSFG Calculations: Empirical Mapping Approach

We have used the well-established electronic structure/molecular dynamics (ES/MD) method[7–9] for the empirical mapping between the quantum mechanical parameters of the oscillator (i.e. the O–H bonds of the water molecules serving as usual as the VSFG chromophore to probe aqueous interfaces) required to calculate the nonlinear spectral lineshape function and the electric field component experienced by the surrounding environment. In this approach, a large number of finite clusters is randomly sampled from a long simulation trajectory of bulk water using the same water model as for the inhomogeneous aqueous systems. Each cluster contains a central O–H bond, providing the vibration of our interest, and all water molecules around that oscillator whose oxygen sites reside within a spherical cutoff of 4 Å around the H atom of that specific O–H bond. All other water molecules that are present within a distance of half the periodic cubic supercell size from that central H atom are considered as background point charges (partial charges as defined by the SPC/E model) for purely electrostatic QM/MM embedding.

For the transition frequency calculations required within the ES/MD approach, the central O–H bond is stretched from 0.72 to 1.28 Å with an increment of 0.2 Å, keeping as usual the positions of all other atoms fixed. The corresponding interactions and, thus, potential energy surface (PES) is generated from ES calculations using the B3LYP functional together with the 6-311++G** as offered by the Gaussian 09 program package.[10] The k-component of transition dipole vector (m_k) and the ij-component of the transition polarizability tensor (a_{ij}) are related to the dipole derivative (μ') and polarizability derivative (α') as follows,[7–9,11]

$$m_k \simeq \mu' x_{10} (\hat{u} \cdot \hat{k}) \ , \qquad (1)$$

$$a_{ij} \simeq x_{10}[(\alpha'_{\parallel} - \alpha'_{\perp})(\hat{u}\cdot\hat{i})(\hat{u}\cdot\hat{j}) + \alpha'_{\perp}(\hat{i}\cdot\hat{j})] \ , \tag{2}$$

where $\hat{u}$ denotes the unit vector along the O–H bond; the derivative of the dipole moment is evaluated along the O–H displacement vector whereas those of the polarizability tensor are taken parallel $\parallel$ and perpendicular $\perp$ with respect to that vector.

The matrix elements (x_{10}) along the vibrational coordinate between ground and excited state of the particular vibration are directly calculated based on those Morse parameters that are determined by fitting the computed PES curves to Morse oscillators for each cluster configuration. As usually done within this approach,[7–9] the required derivatives μ' and α' are computed by first optimizing the central O–H bond while keeping all other atoms fixed, which allows one to carry out a harmonic frequency calculation for each cluster configuration after replacing the mass of all other H atoms by the deuterium mass to single out the particular normal mode that mainly corresponds to O–H stretching from all other normal modes of the cluster, all involving D motion at much lower frequencies. The μ' and α' derivatives are calculated from the IR and Raman intensities as generated by the Gaussian 09 package. The mapping parameters that are used in the present VSFG calculations as well as the details on their determination and parameterization are given in an earlier publication.[12] Thus, technically speaking, we are analyzing here isotopically very dilute mixtures of HOD in D_2O as usually done in the field, both experimentally and computationally. Only that provides a sufficiently small concentration of HDO molecules to decouple the O–H oscillators of interest from the enormous background due to all O–D oscillators in the sample, which are significantly red-shifted with respect to O–H, thereby turning the few O–H bonds (here a single one) into the VSFG chromophore as usual.

In order to validate our ES/MD-based spectral calculations using the electric field mapping technique, we now compare the VSFG response from the bare HBN-water and GRA-water interface to that of the water-air interface in Fig. S1. The water-air surface has been generated here by removing the HBN and GRA sheets from the decoupled HBN-GRA system XXL and corresponding NVE trajectories have been produced with the

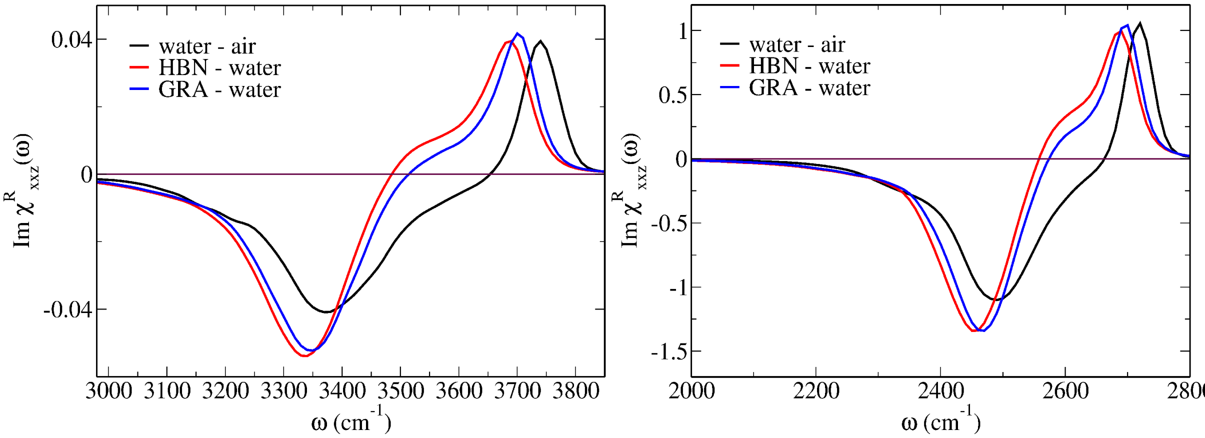

Figure S1: Comparison of the VSFG response from water-air, HBN-water, and GRA-water interfaces for O–H and O–D oscillators in panels (a) and (b), respectively. In panel (b) we applied a similar scaling of the spectral intensity as used previously in Fig. 1(a) of Ref. 13 for the sake of one-to-one comparison. The zero intensity line is shown by a thin solid line.

same procedure as described in Section 5 herein. As depicted in panel (a) of Fig. S1, the VSFG spectra of both interfaces, GRA-water and HBN-water, are red-shifted compared to the water-air interface as is qualitatively expected from the literature.[13] To achieve a more direct comparison with the reported VSFG spectra from electronic structure based *ab initio* molecular dynamics (AIMD) simulations of those surfaces with D_2O, we have calculated the corresponding spectra for O–D oscillators using the present ES/MD technique based on the same simulation trajectories that we have used for all calculations herein. Considering the fact that for the same configuration snapshot the potential energy surfaces of O–H and O–D oscillators are the same within the Born-Oppenheimer treatment, the vibrational frequencies of the corresponding chromophores differ only by their different reduced mass.[15] Using the analogous quadratic relationship of the electric field with frequency as described above for O–H chromophores, we have calculated the VSFG spectra for isotopically diluted O–D oscillators using our simulation trajectories obtained for H_2O, see panel (b) of Fig. S1 for the O–D spectra corresponding to O–H in panel (a). Our VSFG spectra computed using the ES/MD method nicely capture the relative shift in the spectra of GRA-water and HBN-water surfaces as compared to the

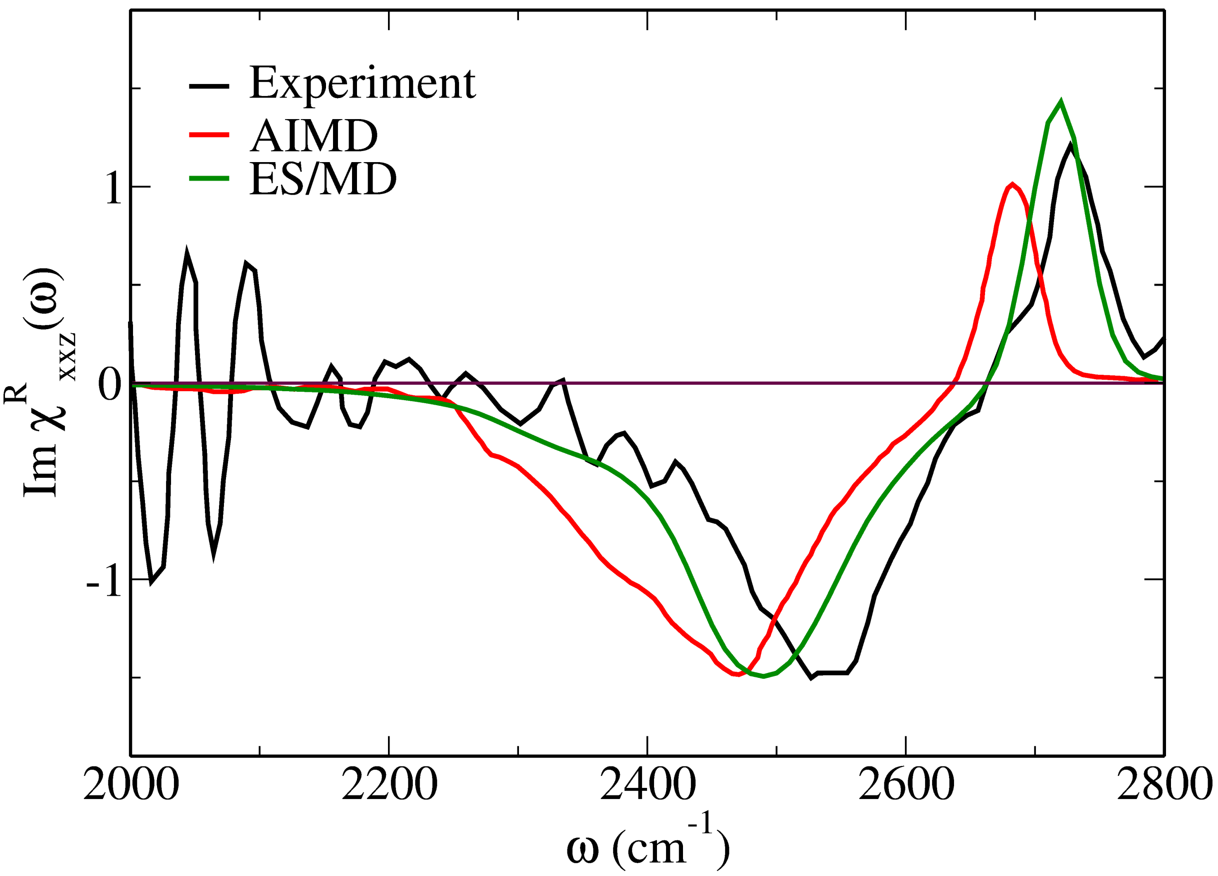

Figure S2: Comparison of the calculated VSFG spectrum of the water-air interface for O–D oscillators as obtained herein using the ES/MD approach to the one computed from electronic structure based *ab initio* molecular dynamics (AIMD) simulations as well as to the experimental spectrum. The shown experimental and AIMD spectra have been digitally extracted from the data published in panel (a) of Fig. 1 of the experimental study[14] and from panel (a) of Fig. 1 of the AIMD investigation,[13] respectively. The zero intensity line is shown by a thin solid line.

water-air surface in substantial accord with earlier *ab initio* VSFG spectra obtained from AIMD simulations .[13] In particular, we refer to panel (a) of Fig. 1 of Ref. 13 for the GRA-water versus water-air VSFG spectrum and to panel (a) of Fig. 3 therein for the direct comparison of HBN-water and GRA-water VSFG spectra. Overall, the corresponding spectral shifts obtained from the parameterized ES/MD technique agree well with those computed using the much more sophisticated *ab initio* MD technique.

In order to finally benchmark both computational approaches with respect to experimental data, we directly compare in Fig. S2 the experimental VSFG spectrum[14] of the

water-air interface of isotopically diluted O–D (i.e. HOD in H_2O) to the calculated spectrum using the ES/MD method (this work) and to the published one based on AIMD simulations.[13] Importantly, both calculations – the parameterized one (ES/MD) and the *ab initio* one (AIMD) – are subject to very different approximations, nevertheless both computed VSFG spectra compare overall equally well to the experimental reference. Note that the experimental spectrum in Ref. 14 has not been corrected by the authors for the Fresnel factor and is, thus, used as reported therein to compare it herein to our computed one – as often done in the VSFG literature,[9,16,17] where the xxz-component of computed spectra are directly compared to the experimental SSP-polarized spectra, as also done here. Based on all these comparisons with respect to the available *ab initio* spectroscopic and experimental VSFG results, we are confident that the spectral fingerprints of the same interfaces but in the slit pore setups as investigated herein using the ES/MD technique are similarly accurate.

Another important way to validate our aforementioned scheme is to check whether symmetric slit pores provide zero response from in our numerical calculations as fundamentally expected. Indeed, in the calculated spectra for the symmetric GRA–GRA monolayer and bilayer slit pores XS and S (see Fig. S3 herein and Fig. 2 in the main text, respectively), no signal beyond the noise is present (see violet circles in both figures).

Furthermore, in an effort to analyze the origin of the zero VSFG signal for the symmetric GRA–GRA monolayer slit pore XS, we have dissected the spectral response in terms of ”up”, ”down” and ”parallel” orientations of the O–H chromophores following the same procedure as used in the decomposition underlying Fig. 3 in the main text (see its panel (a) for the definition of the three orientations) for the asymmetric GRA–HBN monolayer XS, which features a pronounced non-zero VSFG response. Unlike the asymmetric case, up- and down-oriented O–H oscillators within the symmetric XS system produce numerically exactly the same VSFG signal but with opposite sign, which leads to the observed total cancellation that explains the overall zero response from that slit pore.

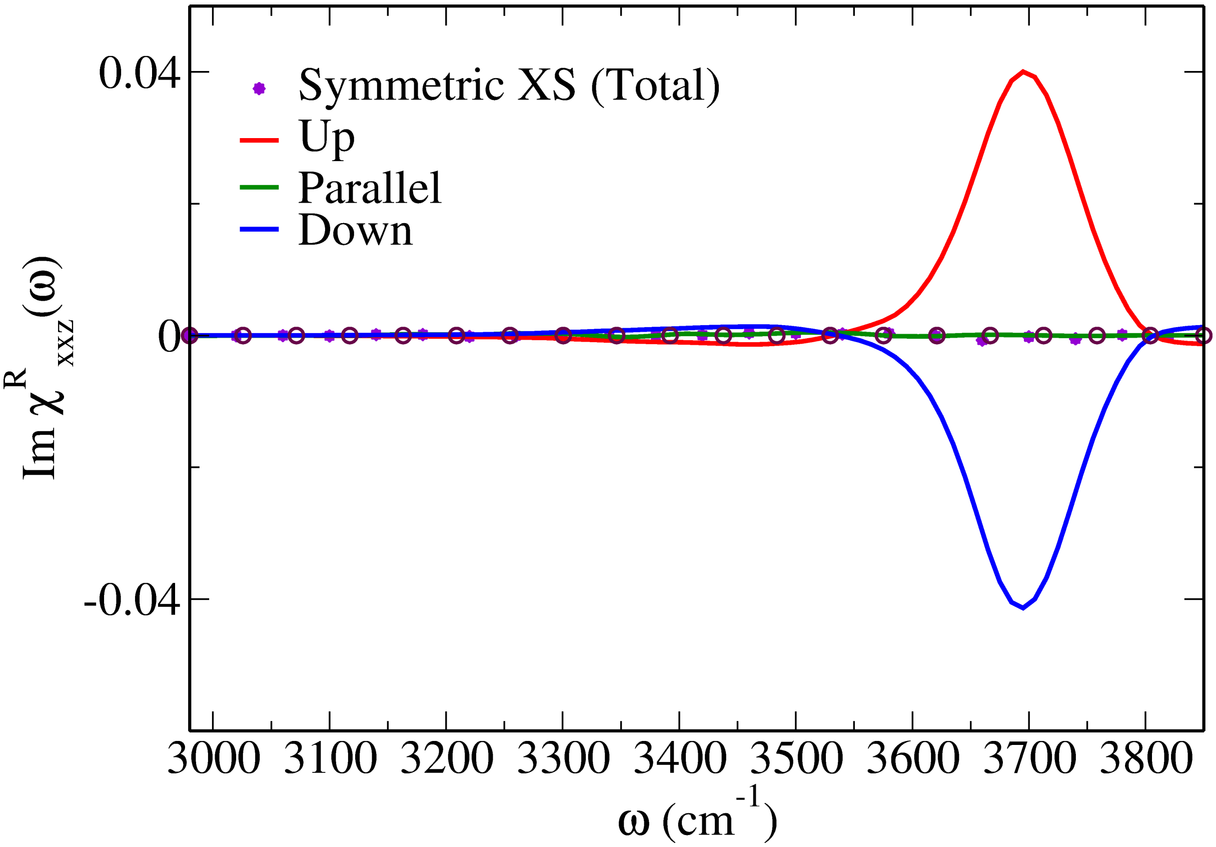

Figure S3: Total VSFG spectrum of the symmetric GRA–GRA monolayer slit pore XS and its decomposition in terms of up, down and parallel orientations of the O–H chromophores following Fig. 3 in the main text. The zero intensity line is shown by open circles.

3. Quantifying Asymmetry in GRA–HBN Slit Pores

The extent of asymmetry of the water lamellae confined within the asymmetric GRA–HBN slit pore setups from XS to XL can be analyzed by comparing the water density along the surface normal with its mirror image. In case of the symmetric GRA–GRA reference slit pores depicted in the right panels of Fig. S4 for the XS to XL systems from top to bottom, we find perfectly symmetrically distributed density profiles with reference to the midpoint of two GRA sheets, thus providing an exact overlap with its mirror image (see the superimposed black and red solid lines) which also demonstrates that our sampling statistics is sufficient. In stark contrast, the minute difference in the interaction strength of water molecules with the HBN versus GRA sheets produces a slight asymmetry of the water density within the GRA–HBN slit pores as depicted in the left panels in Fig. S4. Therefore, even the very mild asymmetry in the water lamellae observed after replacing one GRA wall by HBN (compare the left to the corresponding right panels in that figure) is indeed able to produce finite VSFG responses as shown and discussed in the main text, which notably includes also the monolayer limit in the topmost panel where the asymmetry of the water density is barely visible. Moreover, this finding implies VSFG activity for a large variety of other asymmetric slit pore setups that offer stronger asymmetries due to more pronounced differences in their water-wall interactions and, thus, fruitful experimental prospects.

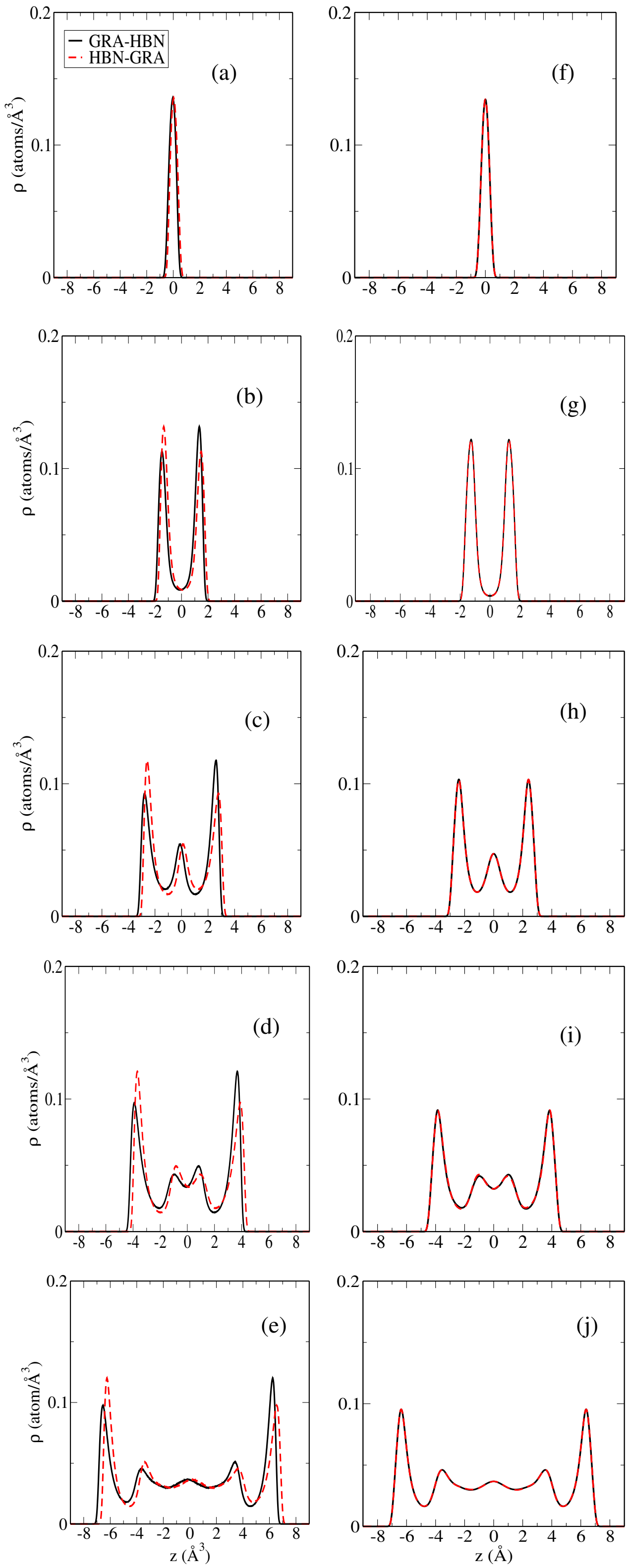

Figure S4: Mirror image comparisons of the density profiles normal to the confining walls, see text, for asymmetric GRA–HBN (left panels) versus symmetric GRA–GRA (right panel) slit pores for the XS, S, M, L, and XL systems from top to bottom.

4. Asymmetric Slit Pores:

Interfacial and Intermediate Solvation Layers

Following the convention of our earlier work on symmetric GRA–GRA slit pores,[1] also in the context of the present asymmetric GRA–HBN systems water is found to be strongly stratified in terms of interfacial (IF) and intermediate (IM) layers depending on the width of the slit pores as depicted in Fig. 1 of the main text. But unlike what happens in the previous symmetric cases, the water density is now slightly tilted towards the HBN surface in case of all GRA–HBN pores. As a result, the different IF and IM layers facing the two walls are no longer equivalent in case of asymmetric planar confinement. Accordingly, the IF layer close to GRA (or HBN) is named IFG (or IFB). Similarly, IMG (or IMB) denote the IM layer close to the GRA (or HBN) wall, whereas the central IM layer being equidistant from HBN and GRA as observed exclusively in the M and XL systems is called IMGB. The spectral decompositions of the total VSFG responses into these various solvation layers are discussed in the main text for the S and XL slit pores (see Fig. 3 therein), whereas the corresponding analyses for the M and L systems are collected in Fig. S5.

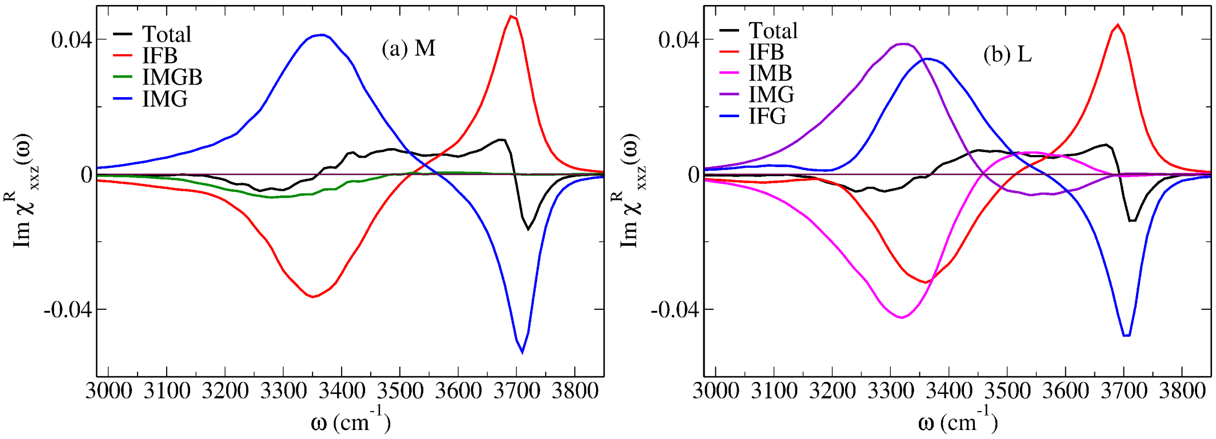

Figure S5: Spectral deconvolution of the total VSFG signal for the M and L slit pores in panels (a) and (b), respectively, in terms of the different interfacial and intermediate solvation layers as explained in the text. The zero intensity line is shown by a thin solid line.

5. Decoupled HBN–Water and GRA–Water Surfaces

In order to calculate the VSFG reference spectra for bare HBN–water and GRA–water interfaces, we have generated a very large system in the slit pore geometry with twice the interlayer distance of the XL slit pore (namely d_{int}= 39.32 Å) as illustrated by Fig. S6(a). It hosts N_{Water} = 1400 water molecules and its interlayer distance is comparable to that of the XXL GRA–GRA slit pore from Ref. 1. The same protocol as described in Sec. has been used to generate 35 statistically independent NVE trajectories that allow us to compute VSFG spectra.

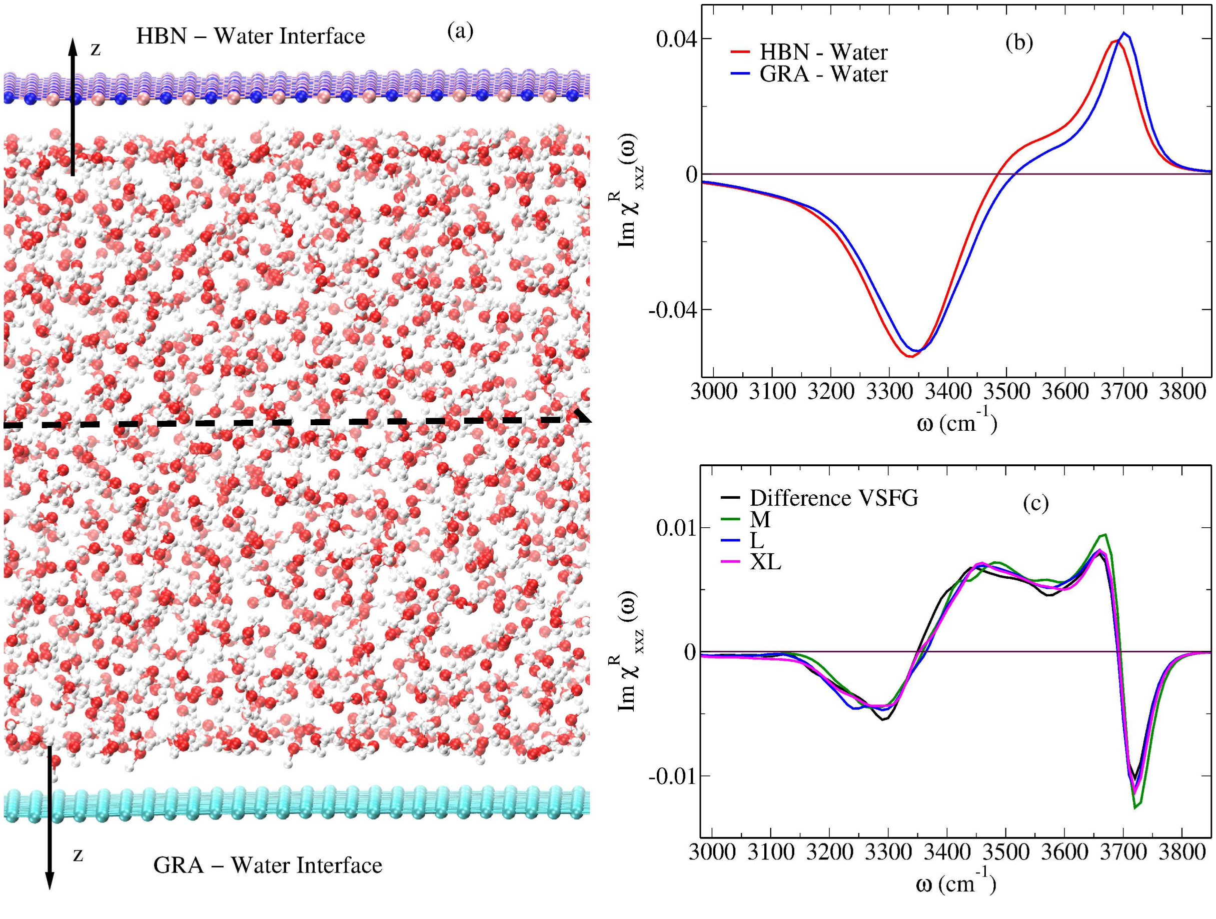

Figure S6: (a) Schematic representation of uncorrelated interfaces, i.e., bare HBN-water and bare GRA-water interfaces, with the hypothetical dividing surface in the middle (black dashed line). (b) VSFG response from the bare interfaces. (c) Comparison of the difference VSFG spectrum form the bare interfaces with the VSFG response from the nanoconfined XL system. The zero intensity line is shown by a thin solid line in panels (b) and (c).

The separate VSFG spectral responses from the bare HBN and GRA interfaces with water can be calculated approximately by assuming a hypothetical dividing surface at at $z = 0$ Å, i.e. equidistantly with respect to the HBN and GRA sheets. The two VSFG responses computed from the water molecules residing above (or below) that dividing surface, i.e. using those with positive (negative) z–values of their O positions, can be considered as the response from the bare HBN–water (or bare GRA–water) interface as reported in Fig. S6(b). Next, the corresponding difference VSFG spectra of such wall–water decoupled interfaces can be calculated by taking the difference of the spectral responses from the bare HBN–water and GRA–water surfaces which is depicted in Fig. S6(c). This difference spectrum serves as our intrinsic reference to probe the (de)coupling of the two interfaces within the XS to XL slit pore setups as detected with VSFG spectroscopy. Comparison with the total VSFG spectra of confined multilayer water as realized by slit pores XL and L down to M shows a negligible coupling of the interfacial water layers close to the HBN and GRA walls since these total spectra are all close to the reference. We conclude that no significant specific confinement effect is seen for these multilayer lamellae.

This is in stark contrast to the total VSFG spectra obtained from the XS and S systems (see Fig. 2(a) of the main text) which significantly deviate from the decoupled reference spectrum. Thus, the strong nanoconfinement effects on the structural dynamics and thus H-bonding of monolayer and bilayer water as a result of significant coupling effects between the HBN and GRA sheets in view of the molecularly small interlayer distances are clearly disclosed by VSFG spectroscopy and assigned as detailed in the main text.

Finally, if we take the difference between the difference VSFG signals from the two bare interfaces, GRA–water and HBN–water as already analyzed in the context of Fig. S6(c), and the VSFG signals from the confined water in asymmetric slit pores, we get double difference VSFG (ddVSFG) spectra which quantitatively disclose the characteristics of the confinement effect. As demonstrated by Fig. S7, the ddVSFG spectrum features

a pronounced finite signal only in case of the monolayer and bilayer slit pores XS and S, whereas this signal is strongly suppressed for moderate to weak confinement (M, L, and XL) yielding multilayered water lamellae instead. Hence, one can use this ddVSFG signal as a fingerprint not only to distinguish but also to characterize the very specific monolayer and bilayer confinement effects with respect to the multilayer cases which are found to merely be (negative) superpositions of the two bare HBN–water and GRA–water interfaces.

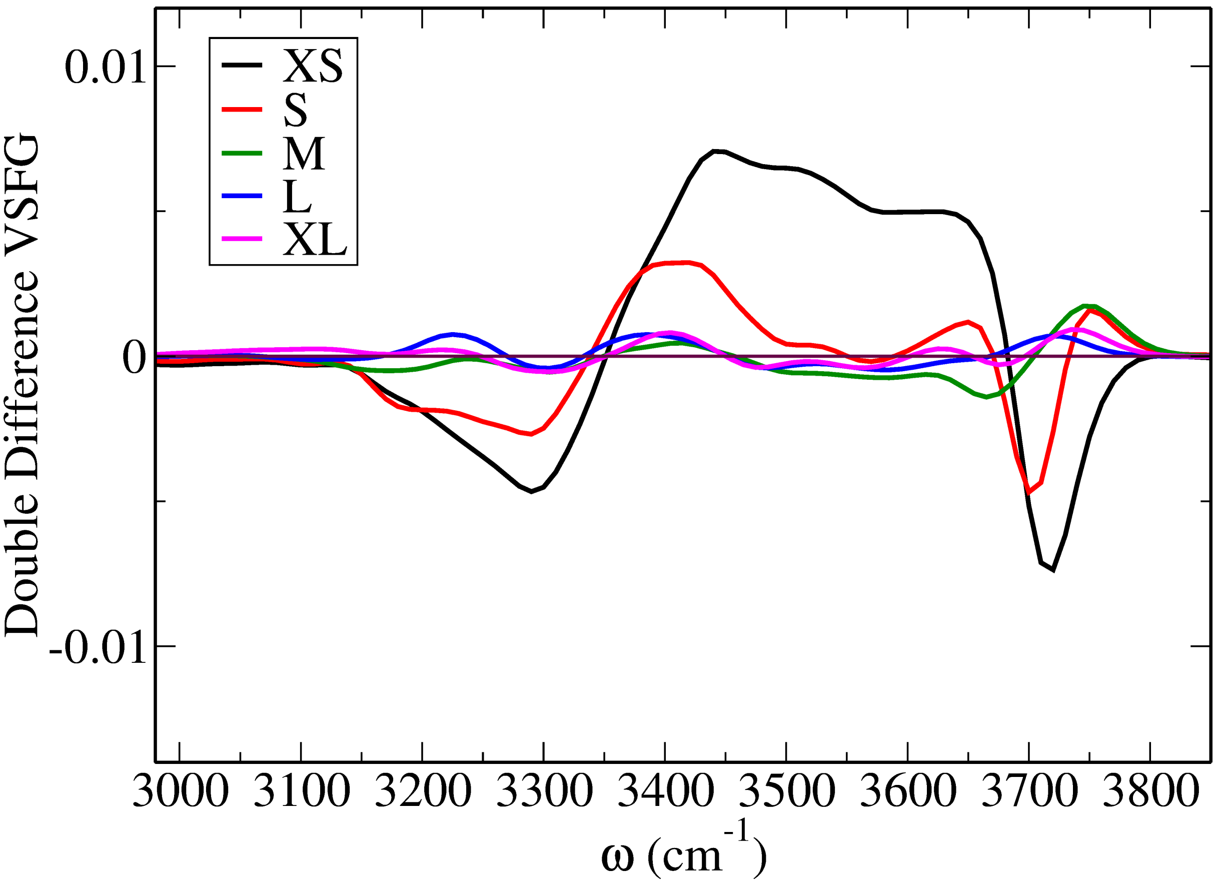

Figure S7: Double difference VSFG spectra, see text, for all asymmetric GRA–HBN slit pores. The zero intensity line is shown by a thin solid line.

6. Spectral Decomposition of VSFG Spectra of Confined Water

Based on the understanding from the rigorous theoretical analyses as discussed in the main text, we now fully outline the proposed spectral decomposition technique based on Lorentzian fittings of spectra to understand the VSFG response from confined water. Lorentzian functions are well known to describe the VSFG spectral lineshapes for bare interfacial water.[9,18] The (complex) Lorentzian function $B_k/(\omega - \omega_k + i\Gamma_k)$ is fully defined by three characteristic parameters, namely the weight B_k related to the spectral density, the center frequency ω_k and the linewidth Γ_k of the k-th oscillator. As shown in Fig. S8 we can fit very well the spectra from both, bare HBN–water and GRA–water interfaces using the following superposition of three Lorentzian functions,

$$\mathrm{Im}\chi^{R}{}_{xxz} = \frac{{B_1}^2}{(\omega-\omega_1)^2+{\Gamma_1}^2} + \frac{{B_2}^2}{(\omega-\omega_2)^2+{\Gamma_2}^2} + \frac{{B_3}^2}{(\omega-\omega_3)^2+{\Gamma_3}^2} \quad . \tag{3}$$

The three Lorentzians correspond to the H–bonded, weakly H–bonded and dangling O–H region for frequencies centered around 3339, 3532 and 3684 cm^{-1} for the HBN–water and 3349, 3557 and 3700 cm^{-1} for the GRA–water interfaces as compiled in Table S2. For both interfaces, the H–bonded oscillators contribute more and have larger linewidth as compared to the dangling (free O–H) oscillators.

Table S2: Lorentzian fitting parameters for the bare HBN–water and GRA–water interfaces, see text.

Interface	${B_1}^2$	ω_1	${\Gamma_1}^2$	${B_2}^2$	ω_2	${\Gamma_2}^2$	${B_3}^2$	ω_3	${\Gamma_3}^2$
HBN–Water	-504.6	3339	8381.3	195.7	3532	10762.0	58.0	3684	1392.3
GRA–Water	-479.8	3349	8399.3	158.7	3557	11539.2	47.2	3700	1042.4

For the confined water VSFG spectra, we have already shown based on detailed theoretical analyses that the overall spectra are shaped as a result of extensive cancellation

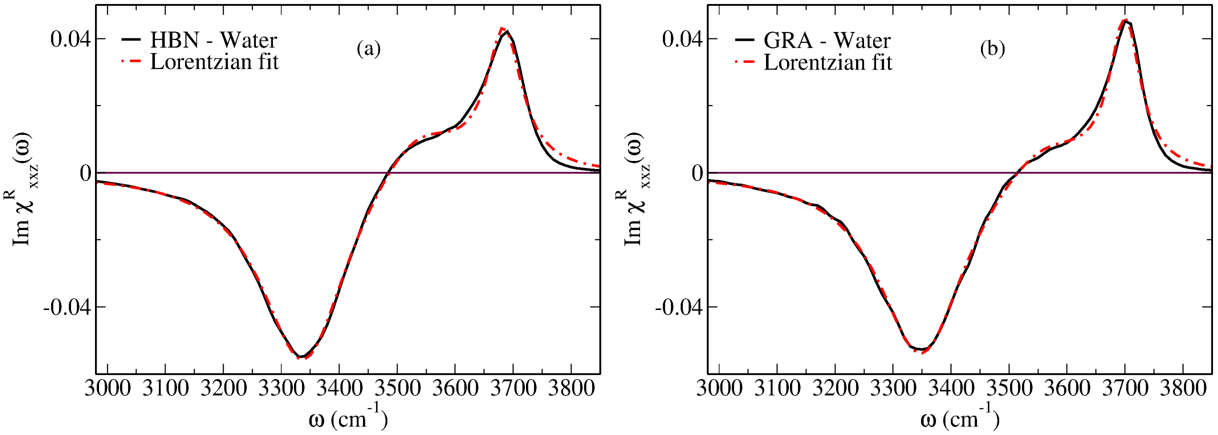

Figure S8: Lorentzian fits of the VSFG spectra of the bare HBN–water and GRA–water interfaces according to Eq. (3) in panels (a) and (b), respectively, in red compared to the corresponding computed spectra in black. The zero intensity line is shown by a thin solid line.

effects of the responses from the two confining surfaces, i.e. HBN–water and GRA–water. Based on that observation, we now introduce a model equation that is the difference between the Lorentzian functions introduced above which characterize the HBN–water (H) and GRA–water (G) interfaces as follows,

$$\begin{aligned}\mathrm{Im}\chi^{R}{}_{xxz} = &\left(\frac{{B_1}^2}{(\omega-\omega_{\mathrm{H1}})^2+{\Gamma_{\mathrm{H1}}}^2}+\frac{{B_2}^2}{(\omega-\omega_{\mathrm{H2}})^2+{\Gamma_{\mathrm{H2}}}^2}+\frac{{B_3}^2}{(\omega-\omega_{\mathrm{H3}})^2+{\Gamma_{\mathrm{H3}}}^2}\right)\\ &-\left(\frac{{B_4}^2}{(\omega-\omega_{\mathrm{G1}})^2+{\Gamma_{\mathrm{G1}}}^2}+\frac{{B_5}^2}{(\omega-\omega_{\mathrm{G2}})^2+{\Gamma_{\mathrm{G2}}}^2}+\frac{{B_6}^2}{(\omega-\omega_{\mathrm{G3}})^2+{\Gamma_{\mathrm{G3}}}^2}\right)\ . \end{aligned} \tag{4}$$

Thus, both the central frequencies and linewidths are considered to be fixed as determined above for the bare interfaces whereas the spectral weights are adjusted to reproduce best the lineshape function computed for the confined systems. As listed in Table S3, the spectral weights for the moderate to weakly confined multilayer systems (M, L, XL) are found to be very similar to those of the the two bare interfaces. This implies that the corresponding VSFG spectra depicted in Fig. S9(b) are very close to the difference VSFG spectrum of the two bare interfaces as already discussed based on Fig. S6(c) where not much confinement effects are found (see main text). In stark contrast, for the bilayer slit pore S the contribution from the H–bonded region is found to be dramatically reduced.

Moreover, in case of the monolayer system XS that contribution it even close to fully suppressed such that the contribution from the dangling O–H region is predominant in the VSFG spectrum. The analysis visualized in panel (a) of Fig. S9 supports and quantifies the qualitative discussion of the very pronounced and distinct confinement effects on water within the very narrow slit pores XS and S in the main text.

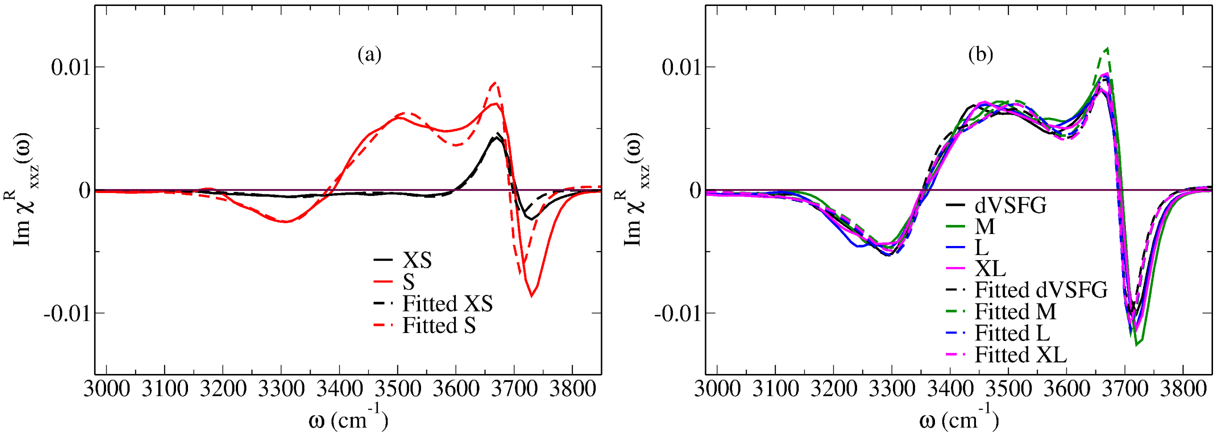

Figure S9: Lorentzian fits of the VSFG spectra of all asymmetric HBN–GRA slit pores. The zero intensity line is shown by a thin solid line.

Table S3: Lorentzian fitting parameters for the asymmetric HBN–GRA slit pores, see text.

Slit Pore	${B_1}^2$	${B_2}^2$	${B_3}^2$	${B_4}^2$	${B_5}^2$	${B_6}^2$
Difference VSFG	-512.6	126.7	42.4	-490.2	78.5	36.3
XL	-442.2	158.9	45.5	-416.3	109.1	38.6
L	-464.1	139.5	44.7	-436.4	84.9	38.7
M	-425.6	169.5	52.5	-400.2	119.0	43.1
S	-114.9	165.0	36.3	-85.3	113.6	28.3
XS	-12.9	20.6	18.0	-8.8	31.8	11.6

Hence, using the introduced fitting technique, one can easily and quantitatively decompose the VSFG spectral lineshape of confined water in terms of the different mechanistic contributions such as H–bonded versus free O–H water molecules. Importantly, this quantitative analysis can also be carried out using purely experimental means – provided only that the VSFG spectra of the two bare interfaces that are used to confine water within the

slit pore setup are known from standard VSFG measurements. The technique ultimately allows one to spectroscopically distinguish the strong confinement effects – as only seen in monolayer and bilayer water lamellae – from multilayer water.

References

(1) Ruiz-Barragan, S.; Muñoz-Santiburcio, D.; Körning, S.; Marx, D. Quantifying anisotropic dielectric response properties of nanoconfined water within graphene slit pores. *Phys. Chem. Chem. Phys.* **2020**, *22*, 10833–10837.

(2) Berendsen, H. J. C.; Grigera, J. R.; Straatsma, T. P. The missing term in effective pair potentials. *J. Phys. Chem.* **1987**, *91*, 6269–6271.

(3) Won, C. Y.; Aluru, N. R. Structure and dynamics of water confined in a boron nitride nanotube. *J. Phys. Chem. C* **2008**, *112*, 1812–1818.

(4) Won, C. Y.; Joseph, S.; Aluru, N. R. Effect of quantum partial charges on the structure and dynamics of water in single-walled carbon nanotubes. *J. Chem. Phys.* **2006**, *125*, 114701.

(5) Hutter, J.; Iannuzzi, M.; Schiffmann, F.; VandeVondele, J. CP2K: atomistic simulations of condensed matter systems. *Wiley Interdiscip. Rev. Comput. Mol. Sci.* **2014**, *4*, 15–25.

(6) CP2K open source molecular dynamics, *https://www.cp2k.org/*

(7) Corcelli, S. A.; Skinner, J. L. Infrared and Raman line shapes of dilute HOD in liquid H_2O and D_2O from 10 to 90 °C. *J. Phys. Chem. A* **2005**, *109*, 6154–6165.

(8) Auer, B.; Kumar, R.; Schmidt, J. R.; Skinner, J. L. Hydrogen bonding and Raman, IR, and 2D-IR spectroscopy of dilute HOD in liquid D_2O. *Proc. Natl. Acad. Sci. U.S.A.* **2007**, *104*, 14215–14220.

(9) Auer, B. M.; Skinner, J. L. Vibrational sum-frequency spectroscopy of the liquid/vapor interface for dilute HOD in D_2O. *J. Chem. Phys.* **2008**, *129*, 214705.

(10) Gaussian09, *http://www. gaussian. com/*

(11) Belch, A. C.; Rice, S. A. The OH stretching spectrum of liquid water: A random network model interpretation. *J. Chem. Phys.* **1983**, *78*, 4817–4823.

(12) Das, B.; Sharma, B.; Chandra, A. Effects of tert-butyl alcohol on water at the liquid–vapor interface: Structurally bulk-like but dynamically slow interfacial water. *J. Phys. Chem. C* **2018**, *122*, 9374–9388.

(13) Ohto, T.; Tada, H.; Nagata, Y. Structure and dynamics of water at water–graphene and water–hexagonal boron-nitride sheet interfaces revealed by *ab initio* sum-frequency generation spectroscopy. *Phys. Chem. Chem. Phys.* **2018**, *20*, 12979–12985.

(14) Ahmed, M.; Nojima, Y.; Nihonyanagi, S.; Yamaguchi, S.; Tahara, T. Comment on Phase-sensitive sum frequency vibrational spectroscopic study of air/water interfaces: H_2O, D_2O, and diluted isotopic mixtures [J. Chem. Phys. 150, 144701 (2019)]. *J. Chem. Phys.* **2020**, *152*, 237101.

(15) Corcelli, S. A.; Lawrence, C. P.; Skinner, J. L. Combined electronic structure/molecular dynamics approach for ultrafast infrared spectroscopy of dilute HOD in liquid H_2O and D_2O. *J. Chem. Phys.* **2004**, *120*, 8107–8117.

(16) Ni, Y.; Skinner, J. L. Communication: Vibrational sum-frequency spectrum of the air-water interface, revisited. *J. Chem. Phys.* **2016**, *145*, 031103.

(17) Tang, F.; Ohto, T.; Sun, S.; Rouxel, J. R.; Imoto, S.; Backus, E. H. G.; Mukamel, S.; Bonn, M.; Nagata, Y. Molecular structure and modeling of water–air and ice–air

interfaces monitored by sum-frequency generation. *Chem. Rev.* **2020**, *120*, 3633–3667.

(18) Montenegro, A.; Dutta, C.; Mammetkuliev, M.; Shi, H.; Hou, B.; Bhattacharyya, D.; Zhao, B.; Cronin, S. B.; Benderskii, A. V. Asymmetric response of interfacial water to applied electric fields. *Nature* **2021**, *594*, 62–65.
